# Multidrug-Resistant Pathogens in Wound Infections: A Systematic Review

**DOI:** 10.7759/cureus.58760

**Published:** 2024-04-22

**Authors:** Faheem Ilyas, Aimen James, Shahid Khan, Soban Haider, Shaukat Ullah, Ghassan Darwish, Syed Ali Hassan Raza Taqvi, Rabia Ali, Qadees Younas, Abdul Rehman

**Affiliations:** 1 Emergency Department, Medcare International Hospital, Gujranwala, PAK; 2 Medicine, Abbottabad International Medical College, Khyber Medical University, Abbottabad, PAK; 3 Medicine, Mission Hospital, Peshawar, PAK; 4 Pediatrics, Ministry of Health, Bisha, SAU; 5 Medical Education and Simulation, Islamic International Medical College, Riphah International University, Rawalpindi, PAK; 6 Zoology, University of Peshawar, Peshawar, PAK; 7 Oral and Maxillofacial Surgery, Faculty of Dentistry, King Abdulaziz University, Jeddah, SAU; 8 Community Medicine, Wah Medical College, Rawalpindi, PAK; 9 General Physician, SHED Hospital, Karachi, PAK; 10 Public Health, Health Services Academy, Islamabad, PAK; 11 Plastic Surgery, Royal College of Surgeons of Edinburgh, Edinburgh, GBR; 12 General Practice, Bolan Medical Complex Hospital, Quetta, PAK

**Keywords:** treatment options, antibiotic resistance, systematic review, wound infections, multidrug-resistant pathogens

## Abstract

This systematic review aimed to explore the antimicrobial activity of a silver-containing gelling fiber dressing against multidrug-resistant organisms (MDROs) in wound infections. It particularly focuses on burn wounds and evaluates its potential clinical significance in combating antimicrobial resistance. A comprehensive literature search was conducted across multiple databases over the past ten years. It is used to identify relevant studies addressing MDRO infections in wound care and exploring novel antimicrobial approaches. The included studies underwent rigorous methodological assessment. Additionally, the data were synthesized to evaluate the efficacy of silver-containing dressings in inhibiting MDRO growth and eradicating biofilm-associated bacteria.

Moreover, this review revealed that silver-containing dressings have constant in vitro antimicrobial activity against 10 MDROs over seven days in simulated wound fluid. However, inhibitory and bactericidal effects were consistently observed against free-living and biofilm phenotypes. The findings suggest potential clinical significance in managing MDRO infections in wounds. This highlights its role in mitigating treatment failure and antimicrobial resistance. Despite the promising implications for wound management practices, this study acknowledges some limitations. In vitro models and the absence of direct clinical validation have also been included.

However, the review explains the importance of new approaches. Nanotechnology has been used to address antimicrobial resistance in wound care. Thus, further research and innovation are needed to improve patient outcomes and combat antimicrobial resistance.

## Introduction and background

Wound infections face a significant challenge in healthcare settings [1]. Specifically, multidrug-resistant organisms (MDROs) are involved [2]. Therefore, wound infections remain a persistent public health concern [3]. Moreover, wound infection leads to prolonged patient debility and increased healthcare costs [4]. Hence, MDROs, such as methicillin-resistant Staphylococcus aureus (MRSA), have emerged and spread [5,6]. In addition, it contains Pseudomonas aeruginosa and Acinetobacter baumannii, which have exacerbated this problem [7]. Therefore, it is a major threat to patient healthcare systems globally.

One of the significant causes of trauma is burn injury [8]. Therefore, it results in compromised skin integrity and increased susceptibility to infection. Patients with burn wounds are especially vulnerable to healthcare-associated infections (HAIs) [9]. It includes various factors, such as loss of the normal skin barrier and impaired immune function. Moreover, it also includes the presence of necrotic tissues. Therefore, these infections contribute significantly to morbidity and mortality rates. Hence, among burn patients, approximately 75% of all deaths related to burn injuries are attributed to infections [10].

Moreover, antibiotic resistance among bacterial pathogens complicates the management of wound infections [11]. This limits treatment options and increases the risk of treatment failure. However, the misuse and overuse of antibiotics have contributed to the rapid emergence of MDROs. These methods make empirical antibiotic therapy less effective and raise concerns about developing novel therapeutic strategies.

Recently, nanotechnology has emerged as a promising approach for improving wound care and combating antibiotic resistance [12]. However, these nanoparticles' unique properties and versatile applications offer new opportunities. These include targeted drug delivery, enhanced antimicrobial activity, and improved wound healing outcomes. By harnessing the potential of nanotechnology, researchers aim to develop innovative wound dressings and treatments [13]. These modalities can address the challenges that MDROs pose in wound infections.

This systematic review consolidates the literature on multidrug-resistant pathogens in wound infections. Additionally, it focuses on burn wounds, prevention strategies, diagnostic methods, and therapeutic approaches. However, we seek to inform clinical practice by examining the incidence, risk factors, microbiological profiles, and antimicrobial susceptibility patterns of MDROs in wound infections [14]. It also guides the development of evidence-based strategies for managing these challenging infections in healthcare settings.

Consequently, our systematic review highlights the pressing need for further research and innovation in managing wound infections caused by multidrug-resistant pathogens [15,16]. However, significant gaps remain in our knowledge and practice despite advancements in understanding the etiology and treatment of such infections. Specifically, there is a lack of comprehensive studies investigating the intricate relationships among host factors, microbial pathogens, and environmental conditions. There is a lack of studies on the development and progression of wound infections, particularly burn injuries. Moreover, the increasing threat of antibiotic resistance among bacterial pathogens underscores the urgency of novel therapeutic approaches. These compounds can effectively target multidrug-resistant organisms while minimizing the risk of treatment failure and antimicrobial resistance [17]. Our study underscores the potential of nanotechnology as a promising avenue for revolutionizing wound care. Therefore, it offers targeted drug delivery [18], enhanced antimicrobial efficacy [19], and accelerated wound healing [20]. However, further research is required to optimize the implementation of nanoparticle-based therapies and their clinical translation. We can advance our understanding of multidrug resistance by addressing these research gaps. The development of evidence-based strategies to improve patient outcomes for wound infections. Finally, the global burden of antimicrobial resistance in healthcare settings can be mitigated.

## Review

Materials and methodology

This study aimed to investigate dressings against multidrug-resistant organisms (MDROs) in both free-living and biofilm states. Hence, stringent in vitro models are used to simulate various wound conditions. The objective of this study was to assess the efficacy of the dressing in inhibiting MDRO growth and eradicating biofilm-associated bacteria. This study provides valuable insights into its potential clinical utility for managing wound infections caused by antibiotic-resistant pathogens.

A comprehensive literature search was conducted by using keywords such as "multidrug-resistant pathogens", "antimicrobial resistance in pathogens", "resistant microbial strains", and "variation" [17]. Therefore, across relevant databases over the past ten years, full-text research articles involving human subjects were included (Table 1).

**Table 1:** Summary of the studies

Database Type	Keywords	Search strategy	Filter Used	No of records
PubMed	Multidrug-resistant pathogens, Microbial resistance	“multidrug-resistant pathogens” OR ”MDR bacteria,” ”antimicrobial resistance in pathogens” AND ”resistant microbial strains,” “microbial resistance in wound infections,” ”antibiotic resistance in wound infections,” ”antibiotic resistance in wound care,” ”drug-resistant wound pathogens” ”emerging resistance in wound bacteria”	Full-text research articles, ten years, humans	498
Scopus	Wound infections	“wound infections,” ”bacterial infections in wounds,” ”types of wound infections,”	Full-text research articles, ten years, humans	482
Google Scholar	Antibiotic resistance	“antibiotic resistance in wound infections,” ”antimicrobial resistance in wound care,” ”bacterial resistance to antibiotics in wounds,”	Full-text research articles, ten years, humans	274

However, the search strategy aimed to identify relevant studies addressing the challenge of MDRO infections in wound care and exploring novel antimicrobial approaches to combat antibiotic resistance. The results showed that the silver-containing dressing sustained in vitro antimicrobial activity against 10 MDROs over seven days in simulated wound fluid. Inhibitory and bactericidal effects were consistently observed. These effects are against free-living and biofilm phenotypes in simulated colonized wound surface models. These findings suggest that silver-containing dressings hold promise as effective interventions for combating MDRO infections in wounds. This shows the threat of antimicrobial resistance while highlighting its potential clinical significance.

A risk of bias assessment was performed. Therefore, we included methodological quality in the assessment. This approach ensures rigorous selection criteria and minimizes potential sources of bias. Hence, the assessment aimed to enhance the validity and reliability of the study findings by critically appraising the design.

The study population comprised MDROs. Moreover, it includes Acinetobacter baumannii, a community-associated methicillin-resistant Staphylococcus aureus (MRSA). Additionally, it has extended the spectrum of beta-lactamase-producing bacteria, and Clostridium difficile has been used to simulate various wound conditions. Furthermore, this study investigated a diverse range of clinically relevant pathogens to provide comprehensive insights. Therefore, silver-containing dressings have antimicrobial effects on common MDROs implicated in wound infections.

The inclusion criteria included full-text articles, systematic reviews, clinical trials, meta-analyses, and observational and experimental studies. These studies focused on wound infections and antibiotic resistance. These studies involved subjects of any age, gender, or ethnicity with documented wound infections caused by multidrug-resistant pathogens [16]. Moreover, interventions or exposures related to wound management, antimicrobial therapies, wound dressings, topical agents, or any interventions addressing wound infections caused by multidrug-resistant pathogens were included. Therefore, the outcome measures included antimicrobial efficacy, infection control, wound healing, antibiotic stewardship, and other relevant clinical outcomes. The review considered studies published in English in the past ten years to ensure relevance to current practices and advancements in wound care and antimicrobial therapies.

Conversely, the exclusion criteria comprised animal studies, conference abstracts, single case reports, or case series. An adequate sample size, non-English publications, and studies unrelated to wound infections, antibiotic resistance, wound management, or antimicrobial therapies are lacking. Moreover, studies focusing on irrelevant interventions, nonhuman subjects, nonoriginal research, or those with a high risk of bias were excluded. However, publications older than ten years were also excluded to ensure alignment with contemporary practices and advancements in wound care and antimicrobial therapies.

Additionally, data were extracted from eligible studies focusing on antimicrobial efficacy, study populations, methodologies, and outcomes related to wound infections and antibiotic resistance [21]. However, this systematic approach facilitated the synthesis and analysis of relevant findings to address the research objectives effectively.

The quality of the included studies was assessed to ensure methodological rigor and validity of the findings. Factors such as study design, sample size, and potential biases were also added. This study aimed to enhance the reliability and robustness of the conclusions drawn from the synthesized data by critically appraising the quality of the evidence (Table 2).

**Table 2:** The mixed methods appraisal tool (MMAT)

Study Title	Citations	Clear Research Question	Appropriate Study Design	Clear Data Collection Method	Well-Established Study Protocol	Satisfactory Response Rate
Multidrug-resistant organisms, wounds, and topical antimicrobial protection	[16]	Yes	Yes	Yes	Yes	N/A (in vitro study)
Multidrug-Resistant Pathogens in Burn Wound, Prevention, Diagnosis, and Therapeutic Approaches	[17]	Yes	Yes	N/A (review article)	N/A (review article)	N/A (review article)
Wound infections	[21]	Yes	Yes	Yes	Yes	N/A (review article)
Burn wound infections	[22]	Yes	Yes	Yes	Yes	N/A (review article)
Antibiotic resistance	[23]	Yes	Yes	Yes	Yes	N/A (review article)
Antibiotic resistance in postoperative infections	[24]	Yes	Yes	Yes	Yes	N/A (review article)
Microbial resistance to drug therapy: a review	[25]	Yes	Yes	Yes	Yes	N/A (review article)
Nanomaterials in Wound Healing and Infection Control	[26]	Yes	Yes	Yes	Yes	N/A (review article)
Treatment strategies for infected wounds	[3]	Yes	Yes	Yes	Yes	N/A (review article)
Therapeutic strategies for chronic wound infection	[27]	Yes	Yes	Yes	Yes	N/A (review article)

The data were synthesized and analyzed to evaluate the overall antimicrobial activity. Silver-containing dressings against MDROs and their potential efficacy in wound management for evaluation were also included. However, this process involved aggregating and interpreting the findings from individual studies to derive meaningful insights into the antimicrobial properties of the dressing, as well as its clinical implications for managing MDRO infections in wounds.

Moreover, ethical considerations were addressed to ensure adherence to ethical guidelines in research involving human subjects and using in vitro models. The study prioritized patient safety, confidentiality, and informed consent by aligning with ethical biomedical research standards.

This study included limitations, such as the use of in vitro models. Without direct clinical validation, these methods may not fully replicate clinical conditions and may not focus on antimicrobial efficacy. The study's reliance on the literature may have introduced inherent biases and limitations associated with retrospective data analysis. These limitations of the study findings were acknowledged to provide a comprehensive and balanced interpretation.

The findings suggest the potential inclusion of silver-containing gelling fiber dressing in protocols aimed at addressing wounds colonized or infected with MDROs, though further research is required. Therefore, it can potentially combat antimicrobial resistance in wound care. Furthermore, the research includes clinical trials and real-world effectiveness studies. Further studies are warranted to validate these findings and translate them effectively into clinical practice.

Results and discussion

**Figure 1:**
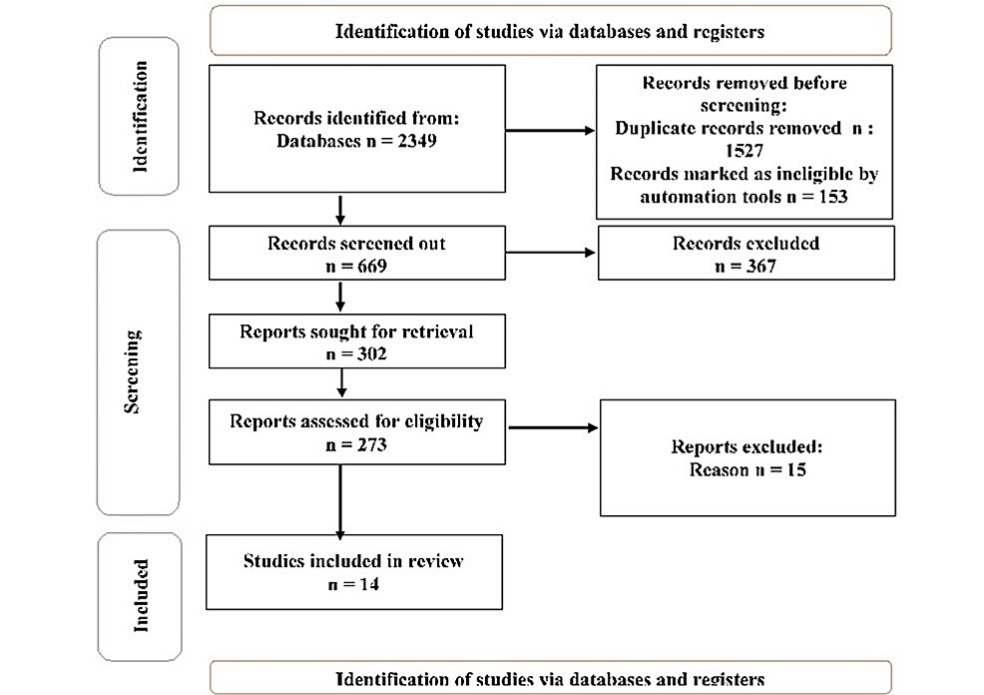
PRISMA flow diagram PRISMA: Preferred reporting items for systematic reviews and meta-analyses

Our systematic review focused on burn wounds and other healthcare-associated infections. However, we synthesized evidence from relevant studies through a comprehensive literature search and rigorous methodological approach (Figure 1). This approach helps to evaluate the efficacy of the dressing in inhibiting MDRO growth and eradicating biofilm-associated bacteria.

Moreover, the results of our study revealed sustained in vitro antimicrobial activity of the silver-containing dressing against ten MDROs over seven days in simulated wound fluid [16]. Thus, inhibitory and bactericidal effects were consistently observed against free-living and biofilm phenotypes in simulated colonized wound surface models. Therefore, these findings underscore the potential of silver-containing dressings as effective interventions for combating MDRO infections in wounds. In particular, the findings highlight settings in which traditional antimicrobial therapies may be limited by antibiotic resistance. A summary of the studies is given (Table 3).

**Table 3:** Summary of the studies

Serial Number	Title	Authors	Findings	Conclusion
1	Multidrug‐resistant organisms, wounds, and topical antimicrobial protection	[16]	Researchers found the antimicrobial activity of silver having gelling fiber dressing against MDROs in free-living and biofilm states. The study findings revealed sustained in vitro antimicrobial activity against 10 MDROs. Inhibitory and bactericidal effects in simulated wound conditions.	The wound protocol involving MDRO is silver-containing gelling and fiber dressing.
2	Multidrug-Resistant Pathogens in Burn Wound, Prevention, Diagnosis, and Therapeutic Approaches	[17]	Highlighted the risk of multidrug-resistant pathogens in burn patients. Emphasized the role of prolonged hospitalization and empirical antibiotics in developing resistance. Explored the use of nanoparticles as an alternative to traditional antimicrobial approaches.	Nanoparticles offer a suitable alternative for treating multidrug-resistant infections in burn wounds.
3	Wound infections	[21]	Identified risk factors for wound infections, including prolonged operative time, preexisting medical illness, and wound contamination. The study explained the importance of antibiotic prophylaxis and wound surveillance in reducing infection rates. s.	Current strategies involve judicious antibiotic use and organized wound surveillance to minimize the infection rate.
4	Burn wound infections	[22]	Discussed classifications, treatment, diagnosis, and prevention of burn wound infections. Highlighted the impact of early excision in decreasing invasive infections. Mentioned the introduction of silver-impregnated devices for reducing nosocomial infections.	Medical advances, including early excision and silver-impregnated devices, have improved outcomes in burn patients.
5	Antibiotic resistance	[23]	Discussed challenges associated with antibiotic resistance in bacterial pathogens. Highlighted the shortage of effective therapies and the involvement of biofilms in multidrug resistance. Covered various resistant organisms, including Staphylococcus aureus and Clostridium difficile.	Urgent need for novel treatment options and alternative antimicrobial therapies due to antibiotic resistance.
6	Antibiotic resistance in postoperative infections	[24]	Explored antibiotic resistance in surgical site infections, emphasizing the impact on surgical patients. Discussed the importance of wound surveillance programs for quality monitoring. Noted the decrease in accuracy of programs due to rapid hospital discharge.	The use of antibiotics in the perioperative period is crucial, and wound surveillance programs play a key role in infection control.
7	Microbial resistance to drug therapy: a review	[25]	Reviewed microbial resistance to antimicrobials, covering parasites, bacteria, fungi, and viruses. Discussed resistance mechanisms and the role of biofilms. Included topics like virulence and control in the Emergency Department.	Microbial resistance is complex, involving microorganisms, the environment, and the patient. Strategies and plans are needed to address resistance and develop alternative therapies.
8	Nanomaterials in Wound Healing and Infection Control	[26]	Explored the role of nanomaterials in wound healing, particularly against infectious biofilms. Moreover, it explained the requirement for novel wound dressings with antibacterial and antibiofilm. Moreover, the wound healing properties. Reviewed various nanomaterials, including metallic nanoparticles and liposomes.	Nanomaterials show promise in developing wound dressings with combined antibacterial and wound-healing properties, which are essential for addressing infectious biofilms.
9	Treatment strategies for infected wounds	[3]	Reviewed antibiotic-embedded wound dressings and their limitations. Explored natural products like essential oils and honey for wound dressings. Highlighted recent advances using nanoparticles for wound healing.	Antibiotic-embedded dressings, essential oils, honey, and nanoparticles offer diverse options for treating infected wounds.
10	Therapeutic strategies for chronic wound infection	[27]	Explored the mechanism of wounds and the importance of identifying and preventing infections promptly. Emphasized the principle of debridement in treating chronic wound infections.	Understanding the pathophysiology of chronic wounds and prompt identification and prevention of infections is crucial in therapeutic strategies.
11	The epidemiology of burn wound infections: then and now	[28]	Discussed changes in the etiology, epidemiology, and prevention of burn wound infections over 50 years. Highlighted the evolution in managing thermal injuries and the need for current epidemiologic studies.	Changes in burn wound management have impacted infection rates, but updated epidemiologic studies are needed to understand the current scenario.
12	Epidemiology and microbiology of surgical wound infections	[29]	A study on surgery patients with wound infections was presented, identifying common bacterial pathogens. Noted a high preponderance of aerobic bacteria and highlighted the need for further research on wound infection etiologies.	Surgical wound infections involve common pathogens, and further research is needed to understand infection etiologies for effective prevention.
13	Risk factors and distribution of MDROs among patients with healthcare-associated burn wound infection	[30]	Assessed the incidence, risk factors, microbiological profile, and antimicrobial susceptibility of healthcare-associated burn wound infections. Significant risk factors were found, and common bacterial isolates, including multidrug-resistant organisms, were identified.	Significant risk factors were the total body surface area >35% and length of stay >14 days. MDROs, including Acinetobacter baumannii, were prevalent. Antimicrobial susceptibility patterns guide tailored antibiotic policies.
14	Using bacterial fluorescence imaging and antimicrobial stewardship to guide wound management practices: a case series	[31]	Evaluated bacterial fluorescence imaging for real-time visualization of bacteria in wounds. Demonstrated its impact on antimicrobial stewardship by preventing unnecessary antibiotic use and guiding evidence-based deployment.	Real-time bacterial fluorescence imaging can provide evidence-based antibiotic use, prevent unnecessary prescriptions, and optimize wound management practices. Additional studies are warranted to explore its diagnostic potential further and its impact on antibiotic usage and wound outcomes.

Our findings align with previous research highlighting the challenges posed by MDROs in wound infections [21], especially among burn patients. Therefore, burn injuries, characterized by compromised skin integrity and increased susceptibility to infection, are particularly vulnerable to MDRO-associated healthcare-associated infections (HAIs). Thus, the spread of MDROs, including methicillin-resistant Staphylococcus aureus (MRSA) and Acinetobacter baumannii [5-7], has further exacerbated the burden of wound infections. This will lead to prolonged hospitalization and increased mortality rates among affected patients.

While discussing the antimicrobial efficacy of silver-containing dressings, the review emphasized their potential in wound management, particularly concerning antibiotic-resistant pathogens. However, targeting MDROs in both free-living and biofilm states may help mitigate the risk of treatment failure and antimicrobial resistance. Therefore, it is effective in improving patient outcomes and reducing healthcare-associated costs. Moreover, its sustained antimicrobial activity over seven days suggests potential benefits in promoting wound healing and preventing infection recurrence, particularly in chronic or complex wound cases.

Acknowledging the limitations of this study, including the use of in vitro models, is crucial as they may not completely replicate clinical conditions. Moreover, the focus on antimicrobial efficacy without direct clinical validation highlights the need for further research. This includes clinical trials and real-world effectiveness studies to validate the findings observed in simulated wound environments. In addition, ethical considerations regarding patient safety, confidentiality, and informed consent must be prioritized in future research involving human subjects and in vitro models.

## Conclusions

This systematic review evaluated various studies on wound infections caused by multidrug-resistant organisms. However, the review revealed that all the included studies had clear research questions, appropriate study designs, and established data collection methods. Additionally, the study protocols were well-established across the board. However, since most of the studies were review articles or focused on in vitro experiments, the concept of a response rate was not applicable. Overall, the review concluded that substantial research addresses the challenges posed by multidrug-resistant pathogens in wound infections. These strategies include burn wound prevention, antibiotic resistance, microbial resistance, and therapeutic strategies for infected wounds and chronic wound infections.

Consequently, our systematic review provides valuable insights into the antimicrobial activity of silver-containing gelling fiber dressings against MDROs in wound infections. The findings support its consideration as part of a comprehensive protocol for managing wounds colonized or infected with MDROs. This highlights its potential role in combating antimicrobial resistance in wound care. Furthermore, additional research is warranted for validation. Hence, these findings can be effectively translated into evidence-based clinical practice.
